# Investigating the impact of Glucagon-like peptide-1 therapy on total shoulder arthroplasty outcomes

**DOI:** 10.1007/s00264-026-06984-z

**Published:** 2026-08-20

**Authors:** Peter Chebi, Apurva Choubey, Ryan Rutherford, Joseph Michalski, Marcelline Kemmy, Kristina Casos, Etienne Flamant, Peter Cirrincione, Benjamin Goldberg

**Affiliations:** Department of Orthopaedic Surgery, University of Illinois Chicago, Chicago, USA

**Keywords:** Total shoulder arthroplasty, GLP-1 receptor agonist, Orthopaedic surgery, Obesity, Infection, Diabetes

## Abstract

**Purpose:**

The increasing prevalence of Glucagon-like peptide-1 receptor agonists (GLP-1 RA) has raised concerns about their perioperative impact. Although database studies on GLP-1 RAs in total shoulder arthroplasty (TSA) have been published for diabetic and general TSA patients, results are conflicting, and the effects on patients across various obesity classes remain unexplored. This study aims to fill this gap.

**Methods:**

The PearlDiver database was used to identify patients who underwent primary TSA, grouped by GLP-1 use, obesity status, and obesity class. Post-operative outcomes included 90-day readmission and emergency department (ED) visit rates, medical complications (including thromboembolic, infectious, and renal events), and orthopaedic complications (including revision, periprosthetic joint infection, and instability). Cohorts were matched 10:1 by exact matching on age, sex, Elixhauser comorbidity index, diabetes, hypertension and overall obesity status, and multivariable regression was used to adjust for comorbidities that remained imbalanced after matching.

**Results:**

Among 95,566 patients, 1,549 (1.62%) were GLP-1 users. Initially, GLP-1 users exhibited higher pre-operative comorbidity burdens and rates of complications. However, after matching and multivariable adjustment for residual imbalance, no significant differences in medical or orthopaedic outcomes persisted. Notably, GLP-1 users demonstrated significantly lower odds of 90-day ED visits (OR 0.75; *P* < .01) and readmissions (OR 0.71; *P* < .01). Similar trends were observed within each individual obesity class, but none reached statistical significance, most likely reflecting the small size of these subgroups.

**Conclusion:**

After accounting for pre-operative comorbidities, our findings suggest that GLP-1 RA use is not associated with worse surgical outcomes in TSA patients and may be associated with lower post-operative healthcare utilisation, although residual confounding cannot be excluded. Further longitudinal studies are warranted to optimise perioperative GLP-1 RA administration.

**Supplementary Information:**

The online version contains supplementary material available at https://doi.org/10.1007/s00264-026-06984-z.

## Introduction

Obesity affects 40% of Americans and 12% of individuals worldwide. Its prevalence has increased over recent decades, having doubled in adults and quadrupled in adolescents since 1990 [1, 2]. Obesity is a major risk factor for glenohumeral osteoarthritis (OA) through increased metabolic disturbances and chronic low-grade inflammation that contribute to cartilage degeneration [3–6]. This trend is consequential for orthopaedic surgeons because OA is the most common indication for primary joint arthroplasty, and surgical intervention is the primary treatment for advanced disease [7–9].

Body mass index (BMI) and obesity are important considerations during surgical planning because associated comorbidities increase perioperative risk [7]. Pharmacologic anti-obesity medications (AOMs), in addition to lifestyle intervention, have become increasingly used to achieve weight loss [10, 11]. Glucagon-like peptide-1 receptor agonists (GLP-1 RAs), initially developed for type 2 diabetes, have demonstrated efficacy in reducing body weight, improving glycaemic control, and exerting anti-inflammatory effects [12]. Stimulating the GLP-1 receptor is thought to decrease weight by several mechanisms, including reducing appetite and food cravings, increasing satiety, and altering food preference and reward pathways [1]. Liraglutide and semaglutide are commonly used GLP-1 RAs shown to reduce weight and improve metabolic and cardiovascular parameters, as well as favourably affect bone metabolism and bone mineral density [13–16].

Obese and diabetic patients undergoing total shoulder arthroplasty (TSA) experience higher rates of infection, venous thromboembolism, revisions, prolonged operative time, and other post-operative complications [17, 18]. A BMI greater than 40 is frequently regarded as a relative contraindication to elective arthroplasty, and obesity is associated with increased risks of dislocation, deep vein thrombosis, infection, prolonged hospitalisation, and revision surgery [19, 20]. A haemoglobin A1c > 7% is another relative contraindication and may delay elective arthroplasty [21]. GLP-1 RAs improve glycaemic control without causing hypoglycaemia outside hyperglycaemic states and have been associated with reduced major adverse cardiovascular events [22, 23].

Despite the established adverse impact of obesity on TSA outcomes, recent database studies evaluating GLP-1 RA exposure at the time of TSA have yielded conflicting results. Elsabbagh et al. reported no difference in medical complications, revision rates, periprosthetic fracture, or infection among diabetic patients (≈50% obese) using GLP-1 RAs versus non-users [24]. Conversely, Lawand et al. found higher rates of medical complications, including deep vein thrombosis, myocardial infarction, pneumonia, transfusion, and readmission, among GLP-1 RA users with mean BMI 34.1–35.5 [25]. Neither study stratified outcomes by obesity class nor comprehensively reported readmissions or emergency department (ED) returns.

This large retrospective cohort study addresses these gaps by validating prior findings, stratifying GLP-1 RA users by obesity class, performing cost analysis, and extending the timeframe and scope of assessed medical and surgical complications. Given that BMI ≥ 40 is both an indication for GLP-1 RA therapy and commonly used as a surgical contraindication, our study aims to clarify outcomes in this population and inform perioperative prognosis and management [26].

## Materials and methods

We conducted a retrospective cohort study using the de-identified PearlDiver MARINER claims database, which includes records from > 170 million patients with private and public insurance from 2010–2022. Because the database contains only de-identified patient records, this study was exempt from institutional review board review and the requirement for informed consent was waived; the study was conducted in accordance with the principles of the Declaration of Helsinki.

Inclusion criteria comprised patients undergoing primary TSA identified by CPT code 23,472 with ≥ five years of available follow-up. Patients were classified by GLP-1 RA use at the time of surgery, defined as an active GLP-1 RA prescription on the day of surgery. Patients were then matched 10:1 (non-users:users), a ratio selected to preserve sample size, by exact matching on age, sex, Elixhauser comorbidity index (ECI), diabetes, hypertension, and overall obesity status (obese versus non-obese). Other comorbidities were not used as matching variables, for two reasons. First, the ECI is a weighted composite that already incorporates renal failure, chronic pulmonary disease, rheumatoid arthritis and collagen vascular disease, deficiency and blood-loss anemia, alcohol abuse and drug abuse. Second, extending exact matching to the full comorbidity list at a 10:1 ratio would have sharply reduced the number of GLP-1 RA users retained, and with it the power to detect the rare orthopaedic outcomes central to the study question. Residual imbalance was instead addressed in the multivariable models described below. Baseline demographics and comorbidities were recorded, including age, ECI, sex, BMI, osteoporosis, rheumatoid arthritis, chronic kidney disease, diabetes, essential hypertension, chronic obstructive pulmonary disease, anaemia, coronary artery disease, tobacco and alcohol use disorders, and pre-operative opioid use disorder. Ninety-day reimbursement, defined as the payer-allowed amount recorded in the database over the 90 days following surgery, was also captured.

Post-operative outcomes included 30-day medical events (deep vein thrombosis, pulmonary embolism, cardiac arrest, transfusion, pneumonia, urinary tract infection, acute kidney injury) and a pooled “All Medical Complications” outcome; hospital length of stay (LOS), which was analysed within the obese cohort; 90-day ED visits and readmissions; 90-day minor wound and infectious complications (wound dehiscence, haematoma, superficial surgical site infection); and five-year orthopaedic sequelae (TSA revision, articular surface wear, implant loosening, periprosthetic fracture, periprosthetic joint infection, shoulder dislocation, subluxation, and periprosthetic osteolysis). Primary analyses included all patients; secondary analyses were restricted to obese patients and to obesity classes: class I (BMI 30.0–34.9), class II (BMI 35.0–39.9), and class III (BMI ≥ 40.0). Independent 10:1 exact matching was performed within each subgroup, comprising obese patients overall and each individual obesity class, on the same covariate set, rather than by subsetting the primary matched cohort. Covariate balance was therefore established separately within every stratum analysed. All diagnoses were identified using International Classification of Diseases (ICD) diagnostic codes.

The PearlDiver data methodology has been previously described [27, 28]. Univariable and multivariable analyses were performed. Categorical variables were compared with Pearson’s chi-square and reported as counts (percent) with crude odds ratios (OR) and 95% confidence intervals (CI). Continuous variables were compared with independent t-tests and reported as mean ± standard deviation. Multivariable logistic regression for categorical outcomes and linear regression for continuous outcomes (LOS) were performed for matched cohorts, controlling for age, sex, ECI, osteoporosis, rheumatoid arthritis, diabetes, hypertension, and tobacco use disorder. This set was kept deliberately parsimonious to avoid overfitting: several orthopaedic outcomes were rare, and because a single covariate set was applied across all outcome models, additional terms would have reduced the number of events per variable to a level at which coefficients become unstable and paradoxical associations more frequent [29]. Covariates were selected for the magnitude of their baseline imbalance and for their validated association with adverse outcome after orthopaedic surgery [30]. Adjusted ORs with 95% Wald CIs are reported. Statistical significance was set at *P* < 0.05.

## Results

95,566 patients who underwent primary TSA between 2010 and 2022 were included, of which 1,549 (1.62%) were GLP-1 users. Before matching, the GLP-1 group had a significantly higher ECI (Non-users: 4.09 ± 3.22 versus GLP-1 users: 6.89 ± 3.56; *P* < 0.001), incidence of obesity (Non-users: 44.05% versus GLP-1 users: 77.73%; *P* < 0.001), lower 90-day reimbursement (non-users: 1,882.59 ± 2,513.12 versus GLP-1 users: 1,717.18 ± 2,694.81; *P* < 0.05), and higher incidence of most tracked pre-operative comorbidities, importantly a pronounced difference in diabetes (Non-users: 37.32% versus GLP-1 users: 98.13%; *P* < 0.001). Matching balanced the primary covariates but did not eliminate all baseline differences, with the non-GLP-1 use group having 14,095 patients following matching, an average age of 66.97 ± 6.82, and average ECI 6.58 ± 3.34, and GLP-1 use group having 1,428 patients with an average age 66.79 ± 6.81 (*P* = 0.35) and average ECI 6.62 ± 3.39 (*P* = 0.68). Reimbursement was also comparable between the groups (Non-users: 1,839.49 ± 2,630.59 versus GLP-1 users: 1,705.21 ± 2,644.19; *P* = 0.07) (Table 1). Osteoporosis, rheumatoid arthritis, chronic obstructive pulmonary disease, tobacco use, alcohol use disorder, and opioid use disorder remained significantly more prevalent among GLP-1 RA non users, and chronic kidney disease remained significantly more prevalent among GLP-1 RA (all *P* < 0.05). Overall obesity status was balanced by design (76.50% versus 76.54%; *P* = 1.00), but the underlying BMI distribution was not: GLP-1 RA users were under-represented below BMI 30.0 and over-represented at BMI ≥ 40.0 (all *P* < 0.01).

**Table 1:** Pre-operative demographic and comorbidity characteristics of all patients undergoing total shoulder arthroplasty, GLP-1 RA users versus non-users

Characteristic	Unmatched			Matched 10:1		
	No GLP-1 (*n* = 94,017)	GLP-1 (*n* = 1,549)	*P*-value	No GLP-1 (*n* = 14,095)	GLP-1 (*n* = 1,428)	*P*-value
Age, mean ± SD	67.84 ± 7.96	66.37 ± 7.01	** < 0.001**	66.97 ± 6.82	66.79 ± 6.81	0.35
ECI, mean ± SD	4.09 ± 3.22	6.89 ± 3.56	** < 0.001**	6.58 ± 3.34	6.62 ± 3.39	0.68
Reimbursement, $ (mean ± SD)	1,882.59 ± 2,513.12	1,717.18 ± 2,694.81	** < 0.05**	1,839.49 ± 2,630.59	1,705.21 ± 2,644.19	0.07
**Gender, n (%)**						
Female	54,294 (57.75%)	937 (60.49%)	** < 0.05**	8,434 (59.84%)	855 (59.87%)	1.00
Male	39,723 (42.25%)	612 (39.51%)		5,661 (40.16%)	573 (40.13%)	
**BMI, n (%)**						
< 20	2,138 (2.27%)	10 (0.65%)	** < 0.001**	251 (1.78%)	9 (0.63%)	** < 0.01**
20.0–24.9	6,840 (7.28%)	40 (2.58%)	** < 0.001**	840 (5.96%)	38 (2.66%)	** < 0.001**
25.0–29.9	13,701 (14.57%)	163 (10.52%)	** < 0.001**	2,286 (16.22%)	144 (10.08%)	** < 0.001**
30.0–34.9 (Obese I)	16,084 (17.11%)	389 (25.11%)	** < 0.001**	3,601 (25.55%)	348 (24.37%)	0.35
35.0–39.9 (Obese II)	10,953 (11.65%)	387 (24.98%)	** < 0.001**	3,166 (22.46%)	349 (24.44%)	0.10
> 40.0 (Obese III +)	8,932 (9.50%)	457 (29.50%)	** < 0.001**	3,015 (21.39%)	402 (28.15%)	** < 0.001**
Obesity (all classes)	41,410 (44.05%)	1,204 (77.73%)	** < 0.001**	10,782 (76.50%)	1,093 (76.54%)	1.00
**Comorbidities, n (%)**						
Osteoporosis	24,645 (26.21%)	327 (21.11%)	** < 0.001**	3,867 (27.44%)	300 (21.01%)	** < 0.001**
Rheumatoid arthritis	12,745 (13.56%)	207 (13.36%)	0.86	2,392 (16.97%)	181 (12.68%)	** < 0.001**
Chronic kidney disease	24,383 (25.93%)	657 (42.41%)	** < 0.001**	5,455 (38.70%)	600 (42.02%)	** < 0.05**
Diabetes	35,085 (37.32%)	1,520 (98.13%)	** < 0.001**	13,809 (97.97%)	1,399 (97.97%)	1.00
Essential hypertension	83,343 (88.65%)	1,515 (97.81%)	** < 0.001**	13,899 (98.61%)	1,404 (98.32%)	0.44
COPD	31,082 (33.06%)	617 (39.83%)	** < 0.001**	6,254 (44.37%)	545 (38.17%)	** < 0.001**
Anaemia	7,134 (7.59%)	159 (10.26%)	** < 0.001**	1,569 (11.13%)	140 (9.80%)	0.14
Coronary artery disease	39,453 (41.96%)	845 (54.55%)	** < 0.001**	7,877 (55.89%)	773 (54.13%)	0.21
Tobacco use	39,350 (41.85%)	674 (43.51%)	0.20	7,054 (50.05%)	610 (42.72%)	** < 0.001**
Alcohol use disorder	6,759 (7.19%)	67 (4.33%)	** < 0.001**	1,245 (8.83%)	63 (4.41%)	** < 0.001**
Opioid use disorder	4,346 (4.62%)	85 (5.49%)	0.12	1,045 (7.41%)	71 (4.97%)	** < 0.001**

*GLP-1 RA* glucagon-like peptide-1 receptor agonist, *SD* standard deviation, *ECI* Elixhauser Comorbidity Index, *BMI* body mass index, *COPD* chronic obstructive pulmonary disease. Continuous variables are reported as mean ± SD; categorical variables as *n* (%). Reimbursement reflects payer-allowed amounts recorded over the 90 days following surgery and does not represent institutional cost. Patients were matched 10:1 (non-users:users) on the listed demographic and comorbidity factors. Bold P-values indicate *P* < 0.05

With regards to post-operative outcomes, GLP-1 users had significantly greater odds of AKI (crude OR 2.17; 95% CI 1.51–3.11; *P* < 0.001), 90-day ED visits (crude OR 1.20; 95% CI 1.02–1.41; *P* < 0.05), 90-day readmissions (crude OR 4.49; 95% CI 3.47–5.81; *P* < 0.001), revision (crude OR 1.36; 95% CI 1.09–1.71; *P* < 0.001), loosening (crude OR 1.41; 95% CI 1.05–1.89; *P* < 0.05), and PJI (crude OR 2.29; 95% CI 1.46–3.60; *P* < 0.001) before matching. After matching and multivariable adjustment, no significant differences in medical or orthopaedic outcomes persisted, whereas GLP-1 users had significantly lower odds of 90-day ED visits (adjusted OR 0.75; 95% CI 0.62–0.90; *P* < 0.01) and 90-day readmissions (adjusted OR 0.71; 95% CI 0.56–0.93; *P* < 0.01) (Table 2).

**Table 2:** Post-operative outcomes among all patients undergoing total shoulder arthroplasty, GLP-1 RA users versus non-users (unmatched and 10:1 matched cohorts)

Clinical outcome	Unmatched				Matched 10:1					
	No GLP-1 (*n* = 94,017)	GLP-1 (*n* = 1,549)	Univariate OR (95% CI)	*P*-value	No GLP-1 (*n* = 14,095)	GLP-1 (*n* = 1,428)	Univariate OR (95% CI)	P-value	Multivariable OR (95% CI)	*P*-value
Deep vein thrombosis	450 (0.48%)	8 (0.52%)	1.08 (0.54–2.18)	0.98	75 (0.53%)	6 (0.42%)	0.79 (0.34–1.81)	0.71	0.79 (0.34–1.82)	0.57
Cardiac arrest	27 (0.03%)	1 (0.06%)	2.25 (0.31–16.56)	0.94	6 (0.04%)	1 (0.07%)	1.65 (0.20–13.68)	1.00	1.49 (0.18–12.50)	0.71
Transfusion	884 (0.94%)	12 (0.77%)	0.82 (0.46–1.46)	0.59	179 (1.27%)	11 (0.77%)	0.60 (0.33–1.11)	0.13	0.62 (0.33–1.14)	0.12
Pulmonary embolism	359 (0.38%)	5 (0.32%)	0.68 (0.25–1.81)	0.56	77 (0.55%)	3 (0.21%)	0.38 (0.12–1.22)	0.13	0.37 (0.12–1.18)	0.09
Pneumonia	917 (0.98%)	16 (1.03%)	1.06 (0.64–1.74)	0.92	200 (1.42%)	15 (1.05%)	0.74 (0.44–1.25)	0.31	0.75 (0.44–1.28)	0.29
Urinary tract infection	1,682 (1.79%)	32 (2.07%)	1.16 (0.81–1.65)	0.47	363 (2.58%)	29 (2.03%)	0.78 (0.54–1.15)	0.25	0.81 (0.55–1.18)	0.27
Acute kidney injury	878 (0.93%)	31 (2.00%)	**2.17 (1.51–3.11)**	** < 0.001**	263 (1.87%)	28 (1.96%)	1.05 (0.71–1.56)	0.88	1.05 (0.71–1.56)	0.82
All medical complications	4,579 (4.87%)	91 (5.87%)	1.22 (0.98–1.51)	0.08	1,002 (7.11%)	82 (5.74%)	0.80 (0.63–1.00)	0.06	0.80 (0.64–1.02)	0.07
**Hospital visits**										
90-day ED visit	8,345 (8.88%)	162 (10.46%)	**1.20 (1.02–1.41)**	** < 0.05**	1,833 (13.00%)	143 (10.01%)	**0.74 (0.62–0.89)**	** < 0.01**	**0.75 (0.62–0.90)**	** < 0.01**
90-day readmission	908 (0.97%)	65 (4.20%)	**4.49 (3.47–5.81)**	** < 0.001**	908 (6.44%)	65 (4.55%)	**0.69 (0.54–0.90)**	** < 0.01**	**0.71 (0.56–0.93)**	** < 0.01**
**Orthopaedic outcomes**										
Revision	3,704 (3.94%)	82 (5.29%)	**1.36 (1.09–1.71)**	** < 0.001**	713 (5.06%)	68 (4.76%)	0.94 (0.73–1.21)	0.67	0.97 (0.75–1.25)	0.79
Periprosthetic fracture	957 (1.02%)	21 (1.36%)	1.34 (0.86–2.07)	0.24	179 (1.27%)	17 (1.19%)	0.94 (0.57–1.54)	0.90	0.97 (0.59–1.61)	0.91
Articular surface wear	179 (0.19%)	2 (0.13%)	0.68 (0.17–2.73)	0.80	33 (0.23%)	1 (0.07%)	0.30 (0.04–2.18)	0.33	0.32 (0.04–2.35)	0.26
Osteolysis	120 (0.13%)	0 (0.00%)	—	0.30	18 (0.13%)	0 (0.00%)	—	0.35	—	—
Prosthesis loosening	2,045 (2.18%)	47 (3.03%)	**1.41 (1.05–1.89)**	** < 0.05**	365 (2.59%)	37 (2.59%)	1.00 (0.71–1.41)	1.00	1.06 (0.75–1.50)	0.74
Shoulder subluxation	469 (0.50%)	11 (0.71%)	1.43 (0.78–2.60)	0.32	85 (0.60%)	10 (0.70%)	1.16 (0.60–2.24)	0.79	1.23 (0.64–2.38)	0.54
Shoulder dislocation	2,718 (2.89%)	40 (2.58%)	0.89 (0.65–1.22)	0.52	489 (3.47%)	36 (2.52%)	0.72 (0.51–1.01)	0.07	0.75 (0.53–1.06)	0.10
Haematoma	352 (0.37%)	5 (0.32%)	0.86 (0.36–2.09)	0.90	67 (0.48%)	5 (0.35%)	0.74 (0.30–1.83)	0.65	0.74 (0.30–1.85)	0.52
Wound dehiscence	221 (0.24%)	3 (0.19%)	0.82 (0.26–2.58)	0.94	53 (0.38%)	2 (0.14%)	0.37 (0.09–1.53)	0.23	0.38 (0.09–1.56)	0.18
Surgical site infection	710 (0.76%)	16 (1.03%)	1.37 (0.83–2.26)	0.27	159 (1.13%)	14 (0.98%)	0.87 (0.50–1.50)	0.71	0.88 (0.51–1.52)	0.64
Periprosthetic joint infection	533 (0.57%)	20 (1.29%)	**2.29 (1.46–3.60)**	** < 0.001**	148 (1.05%)	16 (1.12%)	1.07 (0.64–1.79)	0.91	1.13 (0.67–1.90)	0.66

*GLP-1 RA* glucagon-like peptide-1 receptor agonist, *OR* odds ratio, *CI* confidence interval, *ED* emergency department. Univariate odds ratios are unadjusted; multivariable odds ratios are adjusted for the matching covariates. Bold values indicate *P* < 0.05. An em dash (—) indicates an odds ratio that was not estimable owing to zero events in one group

Post-operative outcomes in the separately matched obese cohort (10,782 non-GLP-1 users; 1,093 GLP-1 users) showed a similar trend, with GLP-1 users having significantly lower 90-day ED visits (adjusted OR 0.80; 95% CI 0.65–0.97; *P* < 0.05) and 90-day readmissions (adjusted OR 0.70; 95% CI 0.52–0.97; *P* < 0.05). Although GLP-1 use was associated with reduced medical complications in univariate analysis (crude OR 0.75; 95% CI 0.58–0.98; *P* < 0.05), significance was no longer appreciated in the multivariable model. No significance was observed in LOS (Table 3).

**Table 3:** Post-operative outcomes among matched obese patients undergoing total shoulder arthroplasty, GLP-1 RA users versus non-users

Clinical outcome	No GLP-1 (*n* = 10,782)	GLP-1 (*n* = 1,093)	Univariate		Multivariable	
			OR (95% CI)	*P*-value	OR (95% CI)	*P*-value
Deep vein thrombosis	69 (0.64%)	6 (0.55%)	0.86 (0.37–1.98)	0.87	0.88 (0.38–2.04)	0.76
Cardiac arrest	5 (0.05%)	1 (0.09%)	1.97 (0.23–16.91)	1.00	1.66 (0.19–14.53)	0.65
Transfusion	133 (1.23%)	9 (0.82%)	0.66 (0.34–1.31)	0.30	0.73 (0.37–1.44)	0.37
Pulmonary embolism	66 (0.61%)	3 (0.27%)	0.45 (0.14–1.42)	0.23	0.45 (0.14–1.44)	0.18
Pneumonia	159 (1.47%)	10 (0.91%)	0.62 (0.32–1.17)	0.18	0.64 (0.34–1.22)	0.18
Urinary tract infection	300 (2.78%)	21 (1.92%)	0.68 (0.44–1.07)	0.12	0.73 (0.47–1.15)	0.17
Acute kidney injury	224 (2.08%)	22 (2.01%)	0.97 (0.62–1.51)	0.97	1.01 (0.65–1.58)	0.97
All medical complications	821 (7.61%)	64 (5.86%)	**0.75 (0.58–0.98)**	** < 0.05**	0.79 (0.61–1.03)	0.09
**Hospital visits**						
Length of stay (days)	8.84 ± 13.42	8.21 ± 11.15	—	0.28	− 0.73 ± 0.64	0.26
90-day ED visit	1,533 (14.22%)	124 (11.34%)	**0.77 (0.64–0.94)**	** < 0.05**	**0.80 (0.65–0.97)**	** < 0.05**
90-day readmission	754 (6.99%)	52 (4.76%)	**0.66 (0.50–0.89)**	** < 0.01**	**0.70 (0.52–0.97)**	** < 0.05**
**Orthopaedic outcomes**						
Revision	592 (5.49%)	56 (5.12%)	0.93 (0.71–1.23)	0.66	0.93 (0.70–1.24)	0.62
Periprosthetic fracture	151 (1.40%)	12 (1.10%)	0.78 (0.43–1.41)	0.49	0.83 (0.46–1.49)	0.53
Articular surface wear	29 (0.27%)	1 (0.09%)	0.34 (0.05–2.50)	0.43	0.37 (0.05–2.69)	0.32
Osteolysis	17 (0.16%)	0 (0.00%)	—	0.37	—	—
Prosthesis loosening	297 (2.75%)	34 (3.11%)	1.13 (0.79–1.63)	0.56	1.17 (0.81–1.68)	0.40
Shoulder subluxation	69 (0.64%)	8 (0.73%)	1.14 (0.55–2.39)	0.87	1.18 (0.57–2.48)	0.65
Shoulder dislocation	376 (3.49%)	33 (3.02%)	0.86 (0.60–1.24)	0.47	0.89 (0.62–1.28)	0.53
Haematoma	47 (0.44%)	5 (0.46%)	1.05 (0.42–2.64)	1.00	1.02 (0.40–2.57)	0.97
Wound dehiscence	48 (0.45%)	1 (0.09%)	0.20 (0.03–1.49)	0.14	0.20 (0.03–1.49)	0.12
Surgical site infection	133 (1.23%)	13 (1.19%)	0.96 (0.54–1.71)	1.00	0.96 (0.54–1.71)	0.89
Periprosthetic joint infection	122 (1.13%)	15 (1.37%)	1.22 (0.71–2.09)	0.57	1.27 (0.73–2.18)	0.40

*GLP-1 RA* glucagon-like peptide-1 receptor agonist, *OR* odds ratio, *CI* confidence interval, *ED* emergency department, *SD* standard deviation. Univariate odds ratios are unadjusted; multivariable odds ratios are adjusted for the matching covariates. Bold values indicate *P* < 0.05. Length of stay is reported as mean ± SD days; its multivariable estimate is a linear regression coefficient (*β* ± standard error) rather than an odds ratio. An em dash (—) indicates an estimate that was not calculable owing to zero events in one group

No significance was observed in post-operative outcomes among class I obese patients (3,601 non-GLP-1 users, 348 GLP-1 users), class II obese patients (3,166 non-GLP-1 users, 349 GLP-1 users), or class III obese patients (3,015 non-GLP-1 users, 402 GLP-1 users), although GLP-1 users continued to show directionally lower, but statistically non-significant, rates of medical complications, 90-day ED visits, and 90-day readmissions (Supplementary Tables 1–3).

## Discussion

In this large retrospective claims analysis of 95,566 primary TSAs, GLP-1 RA users (*n* = 1,549) presented with higher baseline comorbidity burden. Initial unadjusted comparisons suggested increased complication rates among GLP-1 users; however, after matching and multivariable adjustment, GLP-1 exposure was not associated with worse medical or orthopaedic outcomes, owing to GLP-1 users’ poorer pre-operative health. Following matching, GLP-1 users had significantly lower 90-day ED visits and readmissions.

Secondary analyses for obese patients (10,782 non-GLP-1 users and 1,093 GLP-1 users) produced concordant findings. These trends were weakened when filtering for class I obese patients (3,601 non-GLP-1 users, 348 GLP-1 users), class II obese patients (3,166 non-GLP-1 users, 349 GLP-1 users), or class III obese patients (3,015 non-GLP-1 users, 402 GLP-1 users), likely due to low rates for measured events and inability for multivariable regression to properly model such low event sizes. Nevertheless, this study suggests that perioperative GLP-1 use is not associated with worse surgical outcomes in patients undergoing TSA, regardless of patient BMI or obesity class, and that GLP-1 usage may help reduce post-surgical medical complications, hospital admissions, and ED visits. GLP-1 usage was also not associated with higher 90-day reimbursement, or with longer LOS within the obese cohort.

Our findings contradict those of Lawand et al., who used the TriNetX database to show that patients on GLP-1 experienced poorer outcomes following TSA than patients not on GLP-1 but align with and expand on the findings by Elsabbagh et al., who similarly used the PearlDiver database and showed similar results in diabetic GLP-1 and non-GLP-1 users undergoing TSA [24, 25]. Importantly, our study expanded these results to all TSA patients, obese TSA patients, and TSA patients of various obesity classes, with the added findings of improved post-operative outcomes with regards to reduced healthcare utilisation and medical complications.

As rates of TSA, obesity, diabetes, and GLP-1 RA use continue to rise, surgeons will increasingly encounter patients taking GLP-1 RAs and must know how to optimise arthroplasty outcomes. Given GLP-1 RAs’ efficacy for obesity and diabetes, obese individuals should not be routinely advised to discontinue GLP-1 therapy perioperatively; however, prospective studies are needed to further validate these findings.

Potential drawbacks and contraindications of GLP-1 RA administration should be considered in the context of joint replacement, as data specific to TSA are limited. Several reports note that GLP-1 RAs should be held in the setting of acute kidney injury (AKI); GLP-1 RAs, particularly liraglutide, have been associated with AKI, especially in obese patients [4, 31]. It is notable, however, that our study showed an association with GLP-1 RA usage and AKI only before matching, suggesting that GLP-1 RA are not associated with AKI. While other studies have been concerned with perioperative aspiration and regurgitation and the Society for Perioperative Assessment and Quality Improvement recommends continuing GLP-1 agonists up to the day before surgery, some studies have found similar amounts of hypoglycaemia, nausea, and vomiting in GLP-1 RA users undergoing surgery, and this study was unable to assess these outcomes [22, 26]. And while the American Society of Anesthesiologists once recommended discontinuing GLP-1 RAs pre-operatively, this recommendation was eventually reversed.

Despite these concerns, benefits of perioperative GLP-1 RA continuation may outweigh risks [32]. Two relative contraindications for elective surgery, high BMI and elevated haemoglobin A1c, significantly increase surgical risk. Discontinuing GLP-1 RAs can lead to weight regain (patients may regain up to two-thirds of lost weight within one year of semaglutide withdrawal), increasing surgical risk [33]. Stopping GLP-1 RAs may also worsen glycaemic control; post-operative hyperglycaemia impairs bone and soft-tissue healing and is associated with surgical site infections [34].

Another potential reason for continuing GLP-1 RA usage may be cost of care. Obesity and diabetes, among post-operative complications that require increased hospital stay and additional testing, have been associated with higher costs of care for patients undergoing TSA in the immediate post-operative period, and obesity has also been associated with increased surgical failure and cost in the delayed period [35, 36]. Our study failed to show a difference in 90-day reimbursement associated with perioperative GLP-1 RA usage. The weight loss associated with long-term GLP-1 RA usage may be associated with a lower cost of care for TSA, but studies are needed to verify this.

In the unmatched cohort, GLP-1 RA users incurred lower reimbursement than non-users (1,717.18 ± 2,694.81 versus 1,882.59 ± 2,513.12; *P* < 0.05) despite a heavier comorbidity burden (ECI 6.89 versus 4.09). This is unlikely to reflect a true efficiency of therapy. The difference lost significance after matching (*P* = 0.07), and PearlDiver records payer-allowed reimbursement rather than resource utilisation, so contracted rates vary with payer, region and site of service. Differential payer mix is plausible but cannot be resolved with the variables available; stratification by payer type would settle it. Reimbursement in claims data is therefore better read as a payment pattern than as evidence of economic benefit.

Although direct TSA data are limited, evidence from total knee arthroplasty (TKA) supports the potential benefit of GLP-1 RAs in reducing surgical complications. Magruder et al. reported lower rates of prosthetic joint infection (OR 0.70; *P* < 0.001), readmission (OR 0.71; *P* < 0.001), revision (OR 0.86; *P* = 0.02), and lower 90-day costs with semaglutide in TKA patients [37]. Weight loss with GLP-1 RAs can reduce surgical indications and complications: Jin et al. showed > 7.5% weight loss reduced the risk of total knee replacement in obese adults [38]. Zhu et al. found the GLP-1 RA group had greater weight loss (*P* < 0.001) and lower incidence of knee surgery (*P* < 0.05) [39]. BMI > 30 is associated with need for TSA, as well as higher risks of mechanical failure, longer operative time, and infection in TSA [18, 31].

## Strengths and limitations

A strength of this study is the use of aggregated electronic health record data, permitting a large, comprehensive sample at lower cost. We uniquely examined obese patients and stratified obesity classes.

Limitations include the retrospective design, potential inaccuracies in EHR coding, and inability to determine the exact indication for GLP-1 RA prescriptions (diabetes vs. weight loss), type or route of administration for GLP-1 RA, duration of therapy, or specific indications for each primary or revision TSA. Importantly, the PearlDiver database codes for drug prescriptions, not drug administrations, so this study was unable to distinguish patients who had a GLP-1 prescription which was withheld perioperatively. In addition, GLP-1 medications were not indicated for obesity in the absence of diabetes until 2014, which occurred during the study period [1]. Generalisability is also constrained: 98.1% of GLP-1 RA users were diabetic, and the five-year follow-up requirement restricts the cohort to earlier-generation agents rather than contemporary weight-reduction prescribing. Detailed data on surgical technique, perioperative protocols, and post-operative rehabilitation were unavailable and may have influenced outcomes. Residual confounding is a further limitation. Matching balanced the primary covariates but left seven secondary comorbidities imbalanced, and although each was entered into the multivariable models, regression adjustment should not be assumed to remove confounding as completely as balance achieved by design. Six of these seven imbalances left the non-GLP-1 comparator group the sicker of the two, and tobacco, alcohol and opioid use disorders and chronic obstructive pulmonary disease are each strongly associated with unplanned healthcare utilisation. Residual confounding therefore runs in the direction of our reported reductions in emergency department attendance and readmission, and those findings should be interpreted with corresponding caution. Unmeasured confounders including glycaemic control, duration and adherence of GLP-1 RA therapy, functional status and frailty were unavailable. Within individual obesity classes, confidence intervals were wide and consistently included unity, so the absence of significant differences in these strata reflects limited statistical power rather than demonstrated equivalence. Finally, no patient-reported outcome measures were available, limiting assessment of post-operative function. These limitations highlight the need for prospective, randomised trials to validate causality.

## Conclusion

GLP-1 RA use in the perioperative period was not associated with worse surgical outcomes after TSA and may reduce post-operative healthcare utilisation and medical complications across all TSA patients, including those with obesity and morbid obesity. Given rising obesity and GLP-1 RA use, these findings are clinically relevant and support further longitudinal and prospective study to define optimal dosing, patient selection, and timing of GLP-1 RA administration relative to surgery.

## Supplementary Information

Below is the link to the electronic supplementary material.

ESM 1 (DOCX 17.8 KB)

ESM 2 (DOCX 18.8 KB)

ESM 3 (DOCX 17.8 KB)

## Data Availability

No datasets were generated or analysed during the current study.
