# Supplementary material for: Investigating the impact of Glucagon-like peptide-1 therapy on total shoulder arthroplasty outcomes
Source: Int Orthop. 2026 Aug 20;50(10):2671–9. doi: 10.1007/s00264-026-06984-z (PMC13619677; doi:10.1007/s00264-026-06984-z)
Supplement: Supplementary file 1 [file 264_2026_6984_MOESM1_ESM.docx]

Postoperative outcomes for class 1 obese TSA Patients using GLP-1 RA vs. Non-Users – Table 4.

|  | **Univariate** | | | | **Multivariable** | |
| --- | --- | --- | --- | --- | --- | --- |
| **Clinical Outcomes** | **No GLP1 (n=3,601)** | **GLP1 (n=348)** | **GLP1 OR (95% CI)** | **P-value** | **GLP1 OR (95% CI)** | **P-value** |
| **Deep Vein Thrombosis** | 21 (0.58%) | 1 (0.29%) | 0.49 (0.07-3.66) | 0.74 | 0.51 (0.07-3.78) | 0.51 |
| **Cardiac Arrest** | 2 (0.06%) | 0 (0.00%) | - | 1.00 | - | - |
| **Transfusion** | 34 (0.94%) | 0 (0.00%) | - | 0.12 | - | - |
| **Pulmonary Embolism** | 22 (0.61%) | 0 (0.00%) | - | 0.28 | - | - |
| **Pneumonia** | 45 (1.25%) | 4 (1.15%) | 0.92 (0.33-2.57) | 1.00 | 1.07 (0.38-3.01) | 0.99 |
| **Urinary Tract Infection** | 96 (2.67%) | 8 (2.30%) | 0.86 (0.41-1.78) | 0.82 | 0.88 (0.42-1.83) | 0.73 |
| **Acute Kidney Injury** | 75 (2.08%) | 7 (2.01%) | 0.97 (0.44-2.11) | 1.00 | 1.04 (0.47-2.30) | 0.92 |
| **All Medical Complications** | 250 (6.94%) | 20 (5.75%) | 0.82 (0.51-1.31) | 0.46 | 0.88 (0.55-1.42) | 0.60 |
| **Hospital Visits** |  |  |  |  |  |  |
| **Length of Stay** | 6.17 ± 7.50 | 5.28 ± 5.15 | **-** | 0.29 | -0.78 ± 1.06 | 0.46 |
| **90-day ED Visit** | 552 (15.33%) | 44 (12.64%) | 0.80 (0.58-1.11) | 0.21 | 0.87 (0.62-1.21) | 0.41 |
| **90-day Readmissions** | 229 (6.36%) | 13 (3.74%) | 0.57 (0.32-1.01) | 0.06 | 0.62 (0.35-1.09) | 0.10 |
| **Orthopaedic Outcomes** |  |  |  |  |  |  |
| **Revision** | 212 (5.89%) | 20 (5.75%) | 0.97 (0.61-1.56) | 1.00 | 1.05 (0.65-1.69) | 0.85 |
| **Periprosthetic Fracture** | 65 (1.81%) | 1 (0.29%) | 0.16 (0.02-1.13) | 0.06 | 0.17 (0.02-1.22) | 0.08 |
| **Articular Surface Wear** | 11 (0.31%) | 1 (0.29%) | 0.94 (0.12-7.31) | 1.00 | 1.00 (0.12-7.87) | 1.00 |
| **Osteolysis** | 10 (0.28%) | 0 (0.00%) | - | 1.00 | - | - |
| **Prosthesis Loosening** | 114 (3.17%) | 15 (4.31%) | 1,38 (0.79-2.39) | 0.32 | 1.51 (0.86-2.62) | 0.15 |
| **Shoulder Subluxation** | 32 (0.89%) | 4 (1.15%) | 1.30 (0.46-3.69) | 0.85 | 1.49 (0.52-4.28) | 0.46 |
| **Shoulder Dislocation** | 142 (3.94%) | 12 (3.45%) | 0.90 (0.48-1.58) | 0.76 | 0.93 (0.51-1.70) | 0.82 |
| **Hematoma** | 16 (0.44%) | 0 (0.00%) | - | 0.42 | - | - |
| **Wound Dehiscence** | 12 (0.33%) | 0 (0.00%) | - | 0.57 | - | - |
| **Surgical Site Infection** | 28 (0.78%) | 6 (1.72%) | 2.24 (0.92-5.44) | 0.12 | 2.31 (0.94-5.68) | 0.07 |
| **Prosthetic Joint Infection** | 122 (3.39%) | 15 (4.31%) | 1.55 (0.70-3.46) | 0.40 | 1.70 (0.75-3.83) | 0.20 |
