# Supplementary material for: Investigating the impact of Glucagon-like peptide-1 therapy on total shoulder arthroplasty outcomes
Source: Int Orthop. 2026 Aug 20;50(10):2671–9. doi: 10.1007/s00264-026-06984-z (PMC13619677; doi:10.1007/s00264-026-06984-z)
Supplement: Supplementary file 2 [file 264_2026_6984_MOESM2_ESM.docx]

Postoperative outcomes for class 2 obese TSA Patients using GLP-1 RA vs. Non-Users – Table 5

|  | **Univariate** | | | | **Multivariable** | |
| --- | --- | --- | --- | --- | --- | --- |
| **Clinical Outcomes** | **No GLP1 (n=3,166)** | **GLP1 (n=349)** | **GLP1 OR (95% CI)** | **P-value** | **GLP1 OR (95% CI)** | **P-value** |
| **Deep Vein Thrombosis** | 23 (0.73%) | 3 (0.86%) | 1.18 (0.35-3.97) | 1.00 | 1.14 (0.34-3.83) | 0.84 |
| **Cardiac Arrest** | 2 (0.06%) | 0 (0.00%) | - | 1.00 | - | - |
| **Transfusion** | 44 (1.39%) | 3 (0.86%) | 0.62 (0.19-1.99) | 0.57 | 0.60 (0.18-1.98) | 0.41 |
| **Pulmonary Embolism** | 20 (0.63%) | 0 (0.00%) | - | 0.27 | - | - |
| **Pneumonia** | 42 (1.33%) | 4 (1.15%) | 0.86 (0.31-2.42) | 0.97 | 0.86 (0.30-2.43) | 0.77 |
| **Urinary Tract Infection** | 78 (2.46%) | 9 (2.58%) | 1.05 (0.52-2.11) | 1.00 | 1.06 (0.53-2.15) | 0.86 |
| **Acute Kidney Injury** | 79 (2.50%) | 8 (2.29%) | 0.92 (0.44-1.91) | 0.96 | 0.90 (0.43-1.90) | 0.79 |
| **All Medical Complications** | 242 (7.64%) | 26 (7.45%) | 0.97 (0.64-1.48) | 0.98 | 0.98 (0.64-1.50) | 0.93 |
| **Hospital Visits** |  |  |  |  |  |  |
| **Length of Stay** | 5.88 ± 7.10 | 5.53 ± 6.91 | **-** | 0.68 | -0.47 ± 0.82 | 0.57 |
| **90-day ED Visit** | 479 (15.13%) | 42 (12.03%) | 0.77 (0.55-1.07) | 0.14 | 0.79 (0.56-1.11) | 0.17 |
| **90-day Readmissions** | 212 (6.70%) | 22 (6.30%) | 0.94 (0.60-1.48) | 0.87 | 0.98 (0.62-1.54) | 0.92 |
| **Orthopaedic Outcomes** |  |  |  |  |  |  |
| **Revision** | 214 (6.76%) | 25 (7.16%) | 1.06 (0.69-1.64) | 0.86 | 1.10 (0.71-1.69) | 0.67 |
| **Periprosthetic Fracture** | 51 (1.61%) | 6 (1.72%) | 1.07 (0.46-2.51) | 1.00 | 1.14 (0.48-2.68) | 0.77 |
| **Articular Surface Wear** | 12 (0.38%) | 1 (0.29%) | 0.76 (0.10-5.83) | 1.00 | 0.81 (0.10-6.30) | 0.84 |
| **Osteolysis** | 10 (0.32%) | 0 (0.00%) | - | 1.00 | - | - |
| **Prosthesis Loosening** | 118 (3.73%) | 9 (2.58%) | 0.68 (0.34-1.36) | 0.35 | 0.73 (0.37-1.46) | 0.37 |
| **Shoulder Subluxation** | 23 (0.73%) | 2 (0.57%) | 0.79 (0.18-3.35) | 1.00 | 0.76 (0.18-3.23) | 0.71 |
| **Shoulder Dislocation** | 114 (3.60%) | 13 (3.72%) | 1.04 (0.58-1.86) | 1.00 | 1.07 (0.59-1.93) | 0.82 |
| **Hematoma** | 16 (0.51%) | 2 (0.57%) | 1.13 (0.26-4.96) | 1.00 | 1.12 (0.25-4.93) | 0.88 |
| **Wound Dehiscence** | 14 (0.44%) | 0 (0.00%) | - | 0.42 | - | - |
| **Surgical Site Infection** | 39 (1.23%) | 5 (1.43%) | 1.17 (0.46-2.98) | 0.95 | 1.14 (0.44-2.94) | 0.78 |
| **Prosthetic Joint Infection** | 35 (1.11%) | 8 (2.29%) | 2.10 (0.97-4.56) | 0.10 | 2.07 (0.94-4.56) | 0.07 |
