# Supplementary material for: Investigating the impact of Glucagon-like peptide-1 therapy on total shoulder arthroplasty outcomes
Source: Int Orthop. 2026 Aug 20;50(10):2671–9. doi: 10.1007/s00264-026-06984-z (PMC13619677; doi:10.1007/s00264-026-06984-z)
Supplement: Supplementary file 3 [file 264_2026_6984_MOESM3_ESM.docx]

Postoperative outcomes for class 3 obese TSA Patients using GLP-1 RA vs. Non-Users – Table 6

|  | **Univariate** | | | | **Multivariable** | |
| --- | --- | --- | --- | --- | --- | --- |
| **Clinical Outcomes** | **No GLP1 (n=3,015)** | **GLP1 (n=402)** | **GLP1 OR (95% CI)** | **P-value** | **GLP1 OR (95% CI)** | **P-value** |
| **Deep Vein Thrombosis** | 19 (0.63%) | 5 (1.24%) | 1.20 (0.74-5.35) | 0.29 | 2.08 (0.77-5.63) | 0.15 |
| **Cardiac Arrest** | 3 (0.10%) | 1 (0.25%) | 2.50 (0.26-24.13) | 0.96 | 2.36 (0.24-23.14) | 0.46 |
| **Transfusion** | 38 (1.26%) | 3 (0.75%) | 0.59 (0.18-1.92) | 0.52 | 0.65 (0.19-2.14) | 0.48 |
| **Pulmonary Embolism** | 13 (0.43%) | 2 (0.50%) | 1.15 (0.26-5.14) | 1.00 | 1.18 (0.26-5.32) | 0.83 |
| **Pneumonia** | 59 (1.96%) | 6 (1.49%) | 0.76 (0.33-1.77) | 0.66 | 0.78 (0.33-1.83) | 0.57 |
| **Urinary Tract Infection** | 99 (3.28%) | 12 (2.99%) | 0.91 (0.49-1.67) | 0.87 | 0.96 (0.52-1.77) | 0.89 |
| **Acute Kidney Injury** | 97 (3.22%) | 10 (2.49%) | 0.77 (0.40-1.48) | 0.52 | 0.79 (0.40-1.53) | 0.48 |
| **All Medical Complications** | 279 (9.25%) | 33 (8.21%) | 0.88 (0.60-1.28) | 0.55 | 0.92 (0.63-1.35) | 0.67 |
| **Hospital Visits** |  |  |  |  |  |  |
| **Length of Stay** | 11.17 ± 17.91 | 10.18 14.19 | **-** | 0.41 | -0.32 ± 1.38 | 0.81 |
| **90-day ED Visit** | 471 (15.62%) | 52 (12.94%) | 0.80 (0.59-1.09) | 0.18 | 0.84 (0.62-1.15) | 0.28 |
| **90-day Readmissions** | 246 (8.16%) | 22 (5.47%) | 0.65 (0.42-1.02) | 0.07 | 0.68 (0.43-1.07) | 0.09 |
| **Orthopaedic Outcomes** |  |  |  |  |  |  |
| **Revision** | 193 (6.40%) | 21 (5.22%) | 0.81 (0.51-1.28) | 0.42 | 0.82 (0.52-1.31) | 0.41 |
| **Periprosthetic Fracture** | 44 (1.46%) | 7 (1.74%) | 1.20 (0.54-2.67) | 0.83 | 1.24 (0.55-2.77) | 0.60 |
| **Articular Surface Wear** | 11 (0.36%) | 0 (0.00%) | - | 1.00 | - | - |
| **Osteolysis** | 8 (0.27%) | 0 (0.00%) | - | 1.00 | - | - |
| **Prosthesis Loosening** | 96 (3.18%) | 9 (2.24%) | 0.70 (0.35-1.39) | 0.48 | 0.74 (0.37-1.47) | 0.38 |
| **Shoulder Subluxation** | 25 (0.83%) | 4 (1.00%) | 1.20 (0.42-3.47) | 0.96 | 1.24 (0.43-3.60) | 0.69 |
| **Shoulder Dislocation** | 116 (3.85%) | 16 (3.98%) | 1.04 (0.61-1.77) | 1.00 | 1.09 (0.64-1.86) | 0.76 |
| **Hematoma** | 12 (0.40%) | 4 (1.00%) | 2.52 (0.81-7.84) | 0.21 | 2.60 (0.83-8.15) | 0.10 |
| **Wound Dehiscence** | 23 (0.76%) | 1 (0.25%) | 0.32 (0.04-2.41) | 0.40 | 0.36 (0.05-2.68) | 0.32 |
| **Surgical Site Infection** | 53 (1.76%) | 6 (1.49%) | 0.85 (0.36-1.98) | 0.86 | 0.90 (0.38-2.12) | 0.81 |
| **Prosthetic Joint Infection** | 43 (1.43%) | 6 (1.49%) | 1.05 (0.44-2.48) | 1.00 | 1.12 (0.47-2.66) | 0.80 |
